# Microfabrication of X-ray Optics by Metal Assisted Chemical Etching: A Review

**DOI:** 10.3390/mi11060589

**Published:** 2020-06-12

**Authors:** Lucia Romano, Marco Stampanoni

**Affiliations:** 1 Institute for Biomedical Engineering, ETH Zürich, 8092 Zürich, Switzerland; marco.stampanoni@psi.ch; 2 Paul Scherrer Institut, Forschungsstrasse 111, CH-5232 Villigen, Switzerland; 3 CNR-IMM, Department of Physics, University of Catania, 64 via S. Sofia, 95123 Catania, Italy

**Keywords:** X-ray grating interferometry, catalyst, silicon, gold electroplating

## Abstract

High-aspect-ratio silicon micro- and nanostructures are technologically relevant in several applications, such as microelectronics, microelectromechanical systems, sensors, thermoelectric materials, battery anodes, solar cells, photonic devices, and X-ray optics. Microfabrication is usually achieved by dry-etch with reactive ions and KOH based wet-etch, metal assisted chemical etching (MacEtch) is emerging as a new etching technique that allows huge aspect ratio for feature size in the nanoscale. To date, a specialized review of MacEtch that considers both the fundamentals and X-ray optics applications is missing in the literature. This review aims to provide a comprehensive summary including: (i) fundamental mechanism; (ii) basics and roles to perform uniform etching in direction perpendicular to the <100> Si substrate; (iii) several examples of X-ray optics fabricated by MacEtch such as line gratings, circular gratings array, Fresnel zone plates, and other X-ray lenses; (iv) materials and methods for a full fabrication of absorbing gratings and the application in X-ray grating based interferometry; and (v) future perspectives of X-ray optics fabrication. The review provides researchers and engineers with an extensive and updated understanding of the principles and applications of MacEtch as a new technology for X-ray optics fabrication.

## 1. Introduction

High resolution and high-efficiency diffractive optics have largely been unavailable for hard X-rays where many scientific, technological, and biomedical applications exist. This is due to the long-standing challenge of fabricating high aspect ratio high-resolution micro- and nano-structures.

Fabrication of high-aspect-ratio silicon micro- and nano-structures is a key process in many applications, such as microelectronics [1], microelectromechanical systems [2,3], sensors [4], thermoelectric materials [5], battery anodes [6], solar cells [7], photonic devices [8], and X-ray optics [9]. Microfabrication is usually achieved by reactive ion etching [10], which requires high investment in tools and maintenance. KOH-based wet etching [11,12] has been used for microfabrication in Si at micro- and nano-scale. However, the aspect ratio of etched trenches is limited by the etching rate ratio between different crystallographic orientations and only possible in simple geometries like linear gratings or crossed linear gratings defined by the direction of <111> crystallographic planes of Si. As an alternative approach for fabricating Si microstructures, metal assisted chemical etching [13] has attracted great interest [14] because of its simplicity, low fabrication costs, and ability to generate high aspect ratio nanostructures such as nanowires [15]. Several acronyms were reported for this process—MACE, MAE, MacEtch—since 2015 the community seemed to agree with the common acronym of “MacEtch”, which was firstly introduced by X. Li [16] to distinguish the unique properties with respect of standard wet-etch and dry-etch techniques. Unlike KOH wet-etch [12], the MacEtch process is almost independent of crystal orientation and may be used to create a wide variety of patterns, without suffering of microloading effects of dry-etch. An advantage of the method is the considerable reduction in fabrication costs and complexity with respect to the other techniques. MacEtch fabrication of nanoscale patterns has been successfully applied for synchrotron-based X-ray imaging methods [17,18]. For X-ray grating interferometry imaging, the fabrication of Si microgratings requires sharp vertical profiles, high aspect ratios, high accuracy of pitch size and duty cycle, uniformity over large area, and, finally, the possibility to fill up the Si template with a high X-ray absorbing material [19,20] such as gold [21,22]. These requirements are especially stringent for X-ray medical diagnostics for which extremely large field of view is necessary. Thus gratings require microfabrication on area of many squared centimeters [9], with aspect ratio and pitch size that depend on the used energy, specific design and performances (pitch size in the range of 1–20 µm, aspect ratio in the range of 10–100).

Since its discovery in 2000, by Li et al. [13], MacEtch of silicon has emerged as a new technique capable of fabricating 3D nano- and micro-structures of several shapes and applications [23]: —nano-porous film, nanowires [24], 3D objects [25], trenches, vias [26], micro-fins [27], nano-scale grooves, surface antireflection texturing [28], optoelectronic devices such as solar cells [29] and photodetectors [30], sensor devices [31], X-ray optics—in a few semiconductors substrates: Si [15], Ge [30], poly-Si [32], GaAs [33], β-Ga_2_O_3_ [27], SiC [34], etc.—and different catalysts: Ag, Au, Cu, Pt, and Pd [15]. MacEtch has been developed with a strong controlled vertical directionality with respect to the substrate and successfully applied for producing X-ray zone plates [17,23,35,36,37,38] and diffraction gratings [19,20,37,39,40,41]. In MacEtch, a catalyst layer (e.g., Au) is patterned onto the substrate (e.g., Si) to locally increase the dissolution rate of the substrate material in an etchant solution including a fluoride etchant such as hydrofluoric acid (HF) and an oxidizing agent such as hydrogen peroxide (H_2_O_2_).

To date, no comprehensive review of patterned microstructures by MacEtch exists in the literature. The existing reviews on MacEtch rarely focus on the aspects of X-ray gratings fabrication. This paper provides an extensive overview of the fundamentals and recent developments of MacEtch as well as addressing the research gaps in this field. After an overview about the MacEtch mechanism, we dedicated a particular attention to the conditions (catalyst, additives, and reaction temperature) to ensure the vertical etching of the (100) Si substrates. Then, we described the procedures for gratings fabrication, from pattern design to transfer in the silicon substrate and finally the template filling with a high X-ray absorbing material such as Au. In the last section we discussed the recent applications of Fresnel zone plates and X-ray interferometric gratings fabricated by MacEtch. In the concluding remarks we outlined the major challenges for large-scale MacEtch X-ray optics and the perspectives of MacEtch microfabrication.

MacEtch offers the possibility to fabricate high aspect ratio structures for hard X-ray diffractive optics and opens up new opportunities for high resolution imaging with compact X-ray sources and for synchrotrons and X-ray-free electron lasers with more complex wave front manipulation.

## 2. MacEtch Mechanism

The mechanism of MacEtch has been extensively debated in literature [15] even with controversial interpretations [42]. Etching occurs when the metal (catalyst) patterned Si substrate is immersed in a solution with an etchant (for example HF) and an oxidizer (for example H_2_O_2_). The solution-metal-silicon system constitutes a microscopic electrochemical cell that induces anodic silicon etch.

For the most commonly employed oxidants (H_2_O_2_), the proposed cathodic reactions provide free positive carriers to be transferred to the silicon, according to Equation (1). In the anode reaction, the silicon consumes the positive carriers and is solubilized through oxidation (Equation (2)). The concentration of holes becomes higher in the region surrounding the metal catalyst, where silicon is readily oxidized by HF and forms silicon fluoride.

(1) $$H_{2}O_{2}+2H^{+}\to 2H_{2}O+2h^{+}$$

(2) $$\mathrm{Si}+4h^{+}+6\mathrm{HF}\to {\mathrm{SiF}}_{6}^{2-}+6H^{+}$$

Common oxidizers for MacEtch and their associated cathode reactions are reviewed by Chiappini et al. [43]. A wide variety of metal salts can induce silicon porosification without the addition of any other oxidizer, as their electrochemical potential is sufficiently high to directly inject holes in the valence band of silicon [43]. Several other oxidizing agents have been studied [44], including oxygen [37,45] with the following reaction (Equation (3)):

(3) $$O_{2}+4H^{+}+4e^{-}\to 2H_{2}O.$$

**Figure 1:**
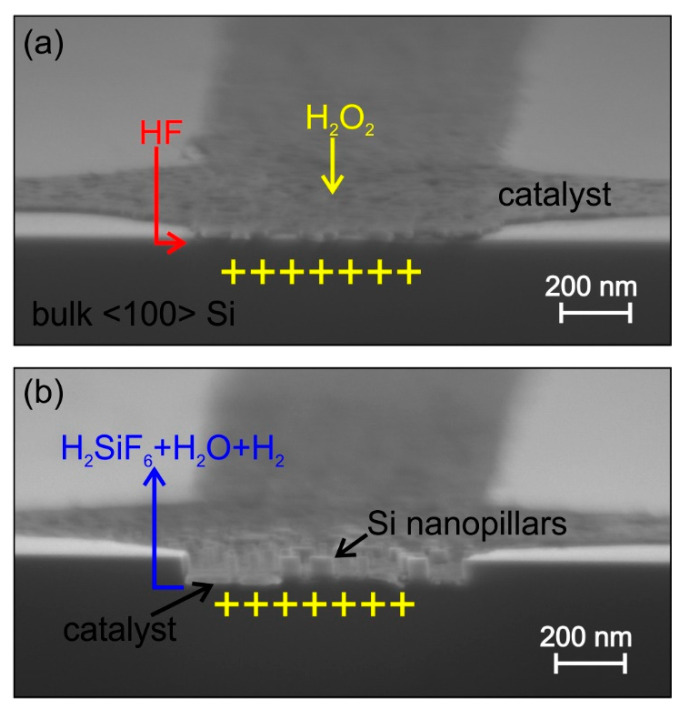
MacEtch mechanism in solution of HF and H_2_O_2_. (**a**) The metal catalyst deposited on a Si <100> substrate decomposes H_2_O_2_ with consequent injection of holes (+) into the semiconductor. (**b**) Si consumes the positive carriers Si, it is readily oxidized by HF and forms silicon fluoride, the process continues and the catalyst progressively sinks into Si along the <100> direction, transferring the nanostructure pattern to the Si (formation of nanopillars in this case). Images are cross-section Scanning Electron Microscopy (SEM) of nano-patterned Pt on Si (**a**) after few seconds of MacEtch in solution of HF and H_2_O_2_.

Figure 1 shows Scanning Electron Microscopy (SEM) images of MacEtch after few seconds of etching. The removal of Si atoms occurs faster at the interface with the metal catalyst, where positive carriers have the maximum concentration. As the reaction proceeds, the catalyst sinks into the substrate and progressively the catalyst nanopattern is transferred to the substrate. The process continues as long as the etchants are present in the solution and the reaction byproducts diffuse out of the pattern. Despite of the simple mechanism, the full process has indeed a complex dynamic where several phenomena—mass transport of etchants and byproducts, charge carrier diffusion, catalyst stability, and gas release—interplay to determine the etching rate, the etching direction, and the quality of the etched structure.

The etching mechanism and the composition dependence have been extensively reported in literature [15,43,46].

Hydrogen peroxide is by far the most commonly employed oxidizer in MacEtch. Chartier et al. assume that the relative concentration of HF and H_2_O_2_ in a MacEtch etch solution plays a similar role to the current density J_ps_ in anodic etch [46]. MacEtch solution is usually described in terms of concentrations ratio between HF and H_2_O_2_, according to Chartier’s formula (Equation (4))

(4) $$\rho =\frac{\left[\mathrm{HF}\right]}{\left[\mathrm{HF}\right]+\left[H_{2}O_{2}\right]}$$

where [HF] and [H_2_O_2_] are the molar concentration of HF and H_2_O_2_, respectively and Hildreth’s [47] compact expression ρ[HF].

## 3. Vertical Etching

**Figure 2:**
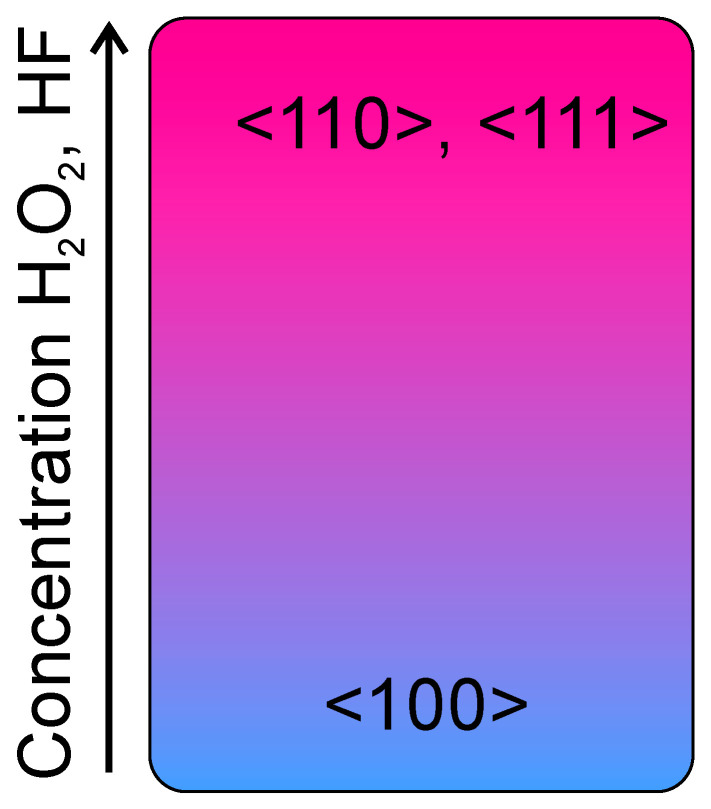
Schematic of preferred etching direction as a function of H_2_O_2_ and HF concentration. The Si back bonds are preferentially removed along the <100> directions in conditions of both low H_2_O_2_ and HF concentrations.

Huang et al. [48] demonstrated that MacEtch is intrinsically anisotropic along the preferred crystallographic <100> directions. Such an orientation dependence is related to the silicon lattice configuration at the reaction site. Removal of oxidized silicon by HF is associated with the cleavage of its back bonds, of which effective number density in different crystal planes increases with the order (100) < (110) < (111) [49]. Due to the different back-bond strength, the Si atom on the (100) surface plane is the most easily removed, and the etching occurs preferentially along the <100> directions. The anisotropy could be reduced or eliminated by varying the concentration of the etchants. In MacEtch of Si, the movement of the etching front (i.e., metal/Si interface) is a net consequence of the following two competing events: (1) injection of a positive charge carriers into bulk Si through the metal-Si interface and (2) removal of oxidized Si by HF from just underneath the catalyst metal. Since the generation of holes is related to the catalytic decomposition of H_2_O_2_ at the interface between the solution and the catalyst metal surface, the amount of holes injected into Si is proportional to the H_2_O_2_ concentration in the solution and the catalyst activity. In conditions of low H_2_O_2_ concentration, hole injection into Si atoms will be localized at the (100) plane, where there are the fewest Si back bonds to break, resulting in etching along the <100> direction. As the concentration of H_2_O_2_ increases sufficiently, removal of oxidized Si would be kinetically favored in the crystal planes with a higher density of silicon back bonds, resulting in etchings along non-<100> directions. The same argument can be played considering HF, in conditions of low HF concentration, the removal of oxidized silicon would control the reaction so the favorite etching direction is again the <100>. While, for high HF concentration, also the other directions are favored. A schematic is reported in Figure 2. It must be noted that H_2_O_2_ and HF are correlated in the solution, so the transition between one etching direction to the other should be determined as a function of the specific solution and the used catalyst. A complex ternary graph would result taking into account the water dilution, as reported by J. Kim et al. [50].

During the process of etching, a triggering event that produces unequal etch rates might occur. These events can change the effective forces on the catalyst and produce a resultant torque on the catalyst. [51]. Such triggering events are made more frequent in the case of higher etch rates as brought about by higher oxidant concentration or by etching at elevated temperature. To date, this represents one of the major challenge to optimize MacEtch as a reliable and controllable process for large area patterning of high aspect ratio structures. Hildreth et al. [25,52] demonstrated that controlled 3D motion of catalyst patterns during MacEtch can be achieved by locally pinning them with an electrically insulating material prior to etching. However, due to this movement, the aspect ratio achievable for features perpendicular to the substrate in an arbitrary dense pattern is limited.

**Figure 3:**
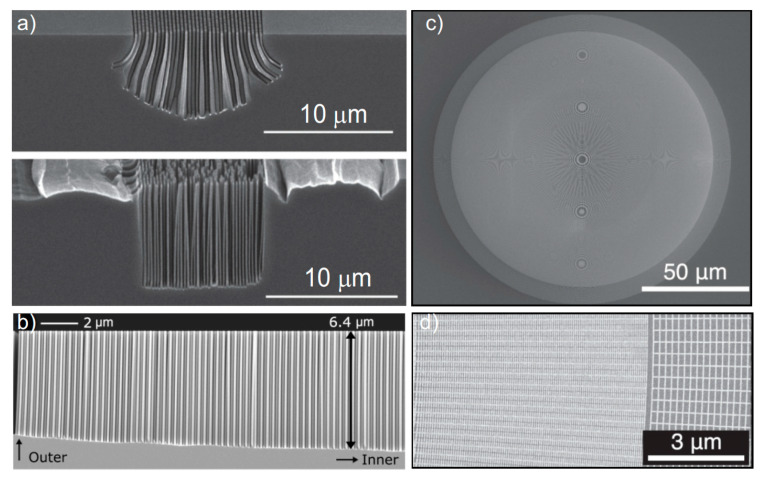
Balancing structures to control the vertical etching at the device border: (**a**) cross section SEM of etched linear grating (pitch 250 nm) with and without balancing structures, the figure was adapted with permission from C. Chang et al., 2014. [17]; (**b**) cross-section SEM of etched kinoform lens with outmost zone of pitch 150 nm, the figure was adapted with permission from M. Lebugle et al., 2018. [41]; (**c**) SEM of Au pattern of zone plates with balancing ring, the figure was adapted with permission from K. Li et al., 2017. [36]; and (**d**) detail of border with balancing ring in (**c**), the figure was adapted with permission from K. Li et al., 2017. [36].

Moreover, charge carriers are injected into Si and charge distribution affects the catalyst movement [53], so that parallel and elongated structures [17] are more difficult to etch than spaced cavities [26]. Several approaches have been attempted in literature to force the uniform etching along the <100> and to minimize the etching along the other directions [51] in order to realize high aspect ratio structures perpendicular to the (100) substrate (vertical etching). Electron-hole concentration balancing structures were used to achieve a vertical etch profile in X-ray zone plates [17]. Figure 3 shows some examples of balancing structures [36] used to define the vertical etching at the borders of the X-ray lens structures [41].

Negative carbon mask [54,55], electrical bias [32,55], and magnetic catalyst [56] have been proposed to force the vertical etching and to improve the control of the catalyst movement [47,52,57]. MacEtch resulted to be very efficient for Si nanostructures, nanowires, and ordered nanopillars [15,29,58,59,60,61], but etching in the microscale regime is more critical [62], the etching rate is limited by the reactant diffusion through the metal mask. The effective transfer of reactants and their by-products would not be identical where the metal pattern size is nanometers or few micrometers. Therefore, two regimes can be distinguished in literature [2,14,63]: (i) nanoscale patterns, in which the etchant species diffuses through the pattern edges and (ii) microscale patterns with nano-porous films, in which the porosity of the film itself controls the diffusion length. In both regimes, the catalyst geometry significantly affects the etching performance. Catalyst optimization and etching conditions are here reviewed in order to address the vertical etching.

### 3.1. Catalyst

A wide range of transition metals can catalyze MacEtch. Noble metals are especially favored for the formation of nanowires as well as for nanostructures with defined cross sections since they better preserve their structure during the etch, as they do not dissolve in HF. Non-noble transition metals have been mostly used to form nano-pores, porous Si, and polished surfaces [43]. The most used MacEthc catalyst is Ag [15]. However, X-ray optics fabrication requires sophisticated patterning techniques such as electron beam lithography or UV photolithography and the catalyst film is usually deposited by thin film evaporation. Silver oxidation is quite difficult to prevent during thin film physical deposition, so Au is the most studied catalyst for thin film deposition. Here, we review the catalyst that have been used for X-ray optics fabrication, which are Au [17,39,40] and Pt [37]. Platinum is the metal with the highest catalytic activity so it allows to obtain the highest MacEtch rate [64]. The patterning of nanostructures requires high precision pattern transfer and high lateral resolution during etching, with MacEtch in liquid this corresponds to a condition of very high HF concentration [17]. Gold catalyst suffers of bad adhesion on silicon substrates, yet a detrimental pattern peel-off has been reported during MacEtch in conditions of high HF concentration [50,65]. On the other hand, uniform high aspect ratio has been reported for nanoporous Au catalyst in conditions of low HF and high H_2_O_2_ concentration [39,66]. In these conditions, the etching is more isotropic [63], the top of the trenches appear wider with respect to the bottom compromising the fidelity of the pattern transfer in the lateral dimension, so the process is not suitable for high aspect ratio structures.

**Figure 4:**
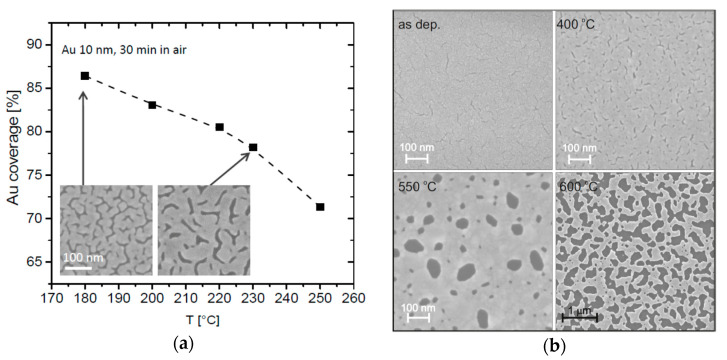
(**a**) Au coverage in percentage of the surface area measured in SEM images in plan-view as a function of the annealing temperature. The Au film thickness was 10 nm and the annealing was performed in air for 30 min. Insets show SEM images of Au film annealed at 180 °C (left) and 230 °C (right), the scale marker is the same in both images. The figure was adapted with permission from L. Romano et al., 2017. [40] (**b**) Pt de-wetting (12 nm) on (100) Si substrate at temperature of 400, 550, and 600 °C. The figure was adapted with permission from L. Romano et al., 2020. [21].

Porous catalyst film is reported [2,63,66,67] to improve the etching performances of micro-scaled Si trenches structures with interconnected catalyst pattern. The porous morphology of the film allows the MacEtch reactants to pass through the catalyst spacing, significantly improving the mass transport and uniformity, which ensures a highly uniform etch rate over all the catalyst area. We recently applied the thermal de-wetting technique to carefully design the film porosity of Au and Pt catalyst and control the vertical etching in micrometer patterns of MacEtch for grating fabrication [21,37,39,40]. Thermal de-wetting is much more robust than evaporation rate to control the film morphology. De-wetting occurs when a thin metal film on a solid substrate is heated, inducing breaking and reassembling of the film [68,69]. The film morphology can be tuned as a function of film thickness and annealing temperature. Figure 4 reports an example of de-wetting for Au film and Pt film deposited on a Si substrate with a cleaned native oxide (oxygen terminated surface). The Pt de-wetting occurs in agreement with literature [69] with a progressive increase of film fractures density (250–350 °C) and finally the hole formation appeared (400–500 °C), followed by a coalescence process of holes expansion (550–600 °C). The thermal treatment in the case of Pt film has two different functions: it creates the porous structure in the metal coating and it forms a platinum silicide at the interface with the substrate that helps to stabilize the catalyst during etching [37].

### 3.2. Alcohols Additives

**Figure 5:**
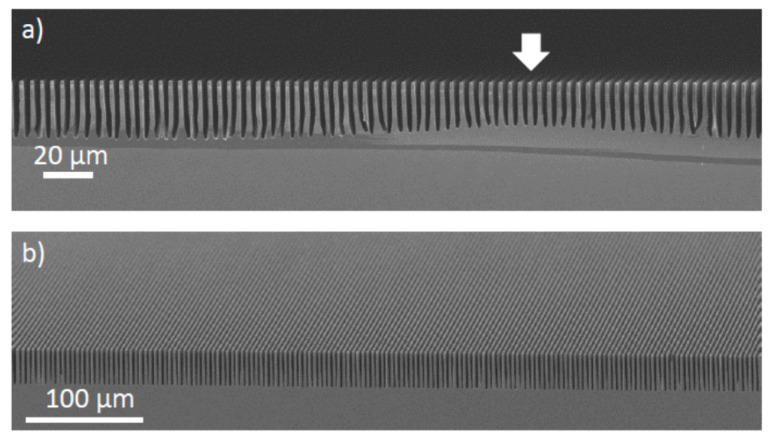
SEM in cross-section of 4.8 µm pitch grating etched with regular MacEtch (**a**) and MacEtch solution with the addition of isopropanol alcohol (**b**). The arrows indicate the presence of a gas bubble preventing the uniform etching of the grating. The figure was adapted with permission from L. Romano et al., 2017. [40].

Ethanol [8] and isopropanol [39] alcohols have been largely used as surfactant in MacEtch solutions. Like in KOH aqueous solutions with addition of alcohol [70], also for MacEtch the alcohol does not take directly part in the etching process, but it strongly affects the etching. Both etch rate and roughness of the etched surface depend on the alcohol concentration in the etching solution, which is connected with the adsorption phenomena on the etched surface [40]. A common issue of MacEtch is the H_2_ gas release during the etching process. The H_2_ is produced as a by-product of reaction [15] and it can substantially affect the etching results since very large bubbles can be formed on the surface of the grating, dramatically preventing a uniform etching. This phenomenon appeared to be much more critical in patterned microstructures than mesh pattern for nanowires since the gas bubbles can be stabilized in the etched structure with liquid solution exhibiting the Cassie-Baxter wetting state [71,72]. The surfactant forms a layer physically covering the surface and prevents the formation of large H_2_ bubbles, reducing the amount and the size of etchant inhomogeneity in contact with the surface [40]. An example of grating fabricated with and without surfactant additive in the etching solution is showed in Figure 5.

**Figure 6:**
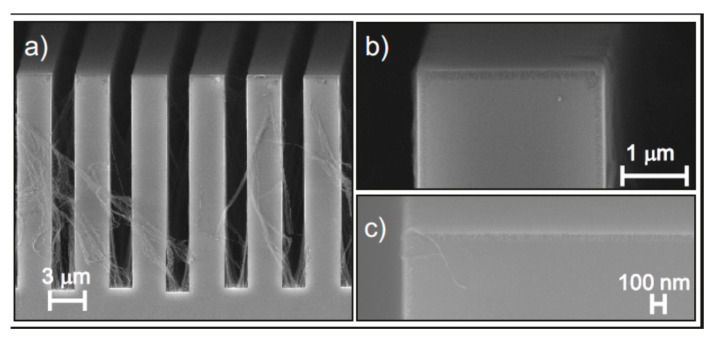
SEM in cross-section of Pt assisted chemical etching of silicon grating with 4.8 µm pitch (**a**,**c**), magnified view of the top (**b**) with etching solution ρ(HF) = 0.99^20^. Magnified view of top (**c**) with additional methanol in the etching solution. The figure was adapted with permission from L. Romano et al., 2020. [21].

Formation of porous Si is a well-known phenomenon which has been observed in MacEtch. The porous morphology of Si using MacEtch has been attributed to the diffusion of holes outside of the metal-semiconductor interface and causing an additional but reduced extent of etching in the areas outside the metal mesh pattern [73]. Depending on etching conditions, pores with different density and thickness can be found at the catalyst/Si interface, along the sidewalls, and within the etched Si nanostructures. In general, a higher oxidant concentration or higher Si doping concentration results in higher levels of porosity. Once the oxidant is reduced on the surface of noble metal, holes are injected into the Si substrate. The holes diffuse from the Si under the noble metal to the off-metal areas that may be etched and form microporous Si. Balasundaram et al. [73] showed that porosity depends on Si doping, the dopant atoms are thermodynamically favorable sites for the formation of pores, and heavily doped Si in liquid MacEtch produces very porous structures even in conditions of very low H_2_O_2_ concentration. The thickness of the microporous Si can be additionally reduced by adding a small amount of alcohol to the etching solution [40]. Figure 6 reports a magnified SEM of the top Si lamellas in Pt-MacEtch with additional methanol, the microporous thickness is less than 50 nm. Methanol is less affecting the etching rate with respect of isopropanol and ethanol alcohols [74].

**Figure 7:**
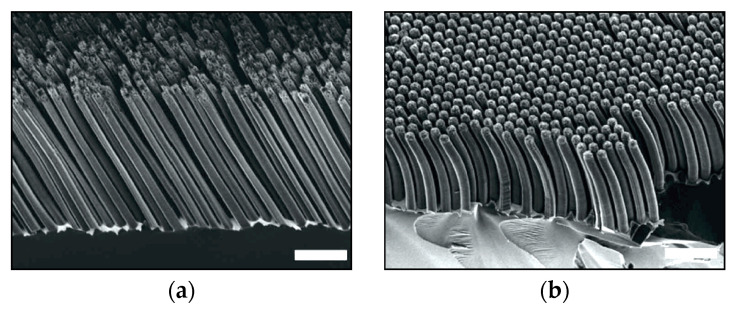
SEM images of Si nanowires etched for 20 min in HF–H_2_O_2_–water–co-solvent (**a**) isopropanol 2:1:5:2 and (**b**) acetonitrile 2:1:5:2. All scale bars are 2 µm. The figure was adapted with permission from Y. Kim et al., 2013. [74].

However, a large amount of additive (methanol, ethanol, isopropanol, and acetonitrile in the HF–H_2_O_2_–H_2_O solution) can cause the changing of the etching direction, inducing the formation of curved or tilted structures [74]. An example is reported in Figure 7, where curved Si nanowires are produced in a solution with isopropanol and acetonitrile. H_2_O_2_ is more severely shielded from reaction sites by the additive than HF is, due to the higher surface tension of H_2_O_2_, effectively increasing the HF to H_2_O_2_ ratio locally.

### 3.3. Temperature

With a temperature in the range of 0 °C to 50 °C, Cheng et al. [75] observed a linear relationship between length of nanowire and etching time. The etching rate increased with increasing etching temperature with an activation energy of 0.36 eV for the formation of Si nanowires on a (100) Si substrate in AgNO_3_ and HF aqueous solution.

**Figure 8:**
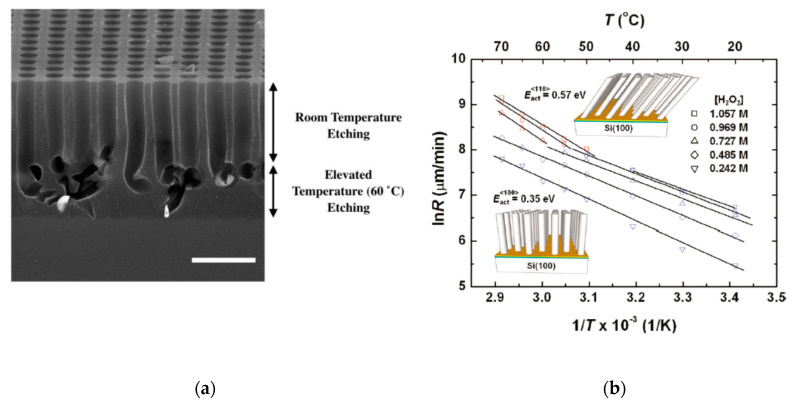
(**a**) Cross-sectional SEM images of the as-etched Si produced with Au catalyst of *p*-type Si (100) substrate (0.005 Ωcm) in solution of 13.5 M HF and 0.16 M H_2_O_2_ at room temperature for 30 min and subsequently etched at the same condition at 60 ˚C for 1 min. Scale bar is 2 μm. The figure was adapted with permission from L. Kong et al., 2017. [51]. (**b**) Temperature dependence of etching rate for different H_2_O_2_ concentration, at high temperature the etching direction changes from (100) to (110). The figure was adapted with permission from J. Kim et al., 2011. [50].

**Figure 9:**
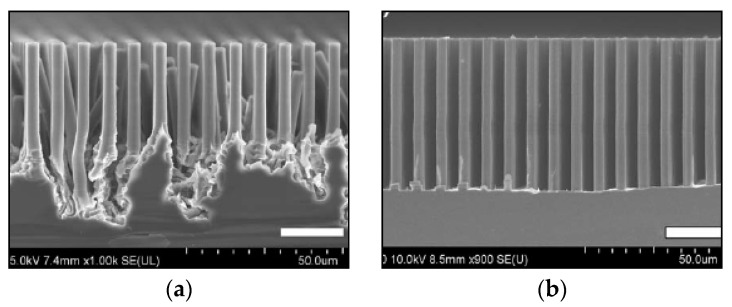
Cross-sectional SEM images of the as-etched Si produced with a catalyst of 40/5 nm thick Au/Ti of n-type Si (100) substrate (0.9–1.1 Ωcm). The etching temperatures are 30 °C (**a**) and 8 °C (**b**), respectively. The etching duration and concentration of H_2_O_2_ are 12 h and 0.2 M, respectively. Scale bar is 20 µm in all figures. The figure was adapted with permission from J. Yan et al., 2016. [76].

Temperature has also a strong effect on the etching direction. Figure 8 shows the effect of temperature on etching direction for (100) substrates, as the temperature increases (50–70 °C) the etching along the (110) instead of (100) is preferred. Figure 9 shows a comparison of etching at 30 °C and 8 °C, indicating the temperature reduction as a possible way to control the vertical etching.

The Si porosity is strongly affected by the etching temperature. Excess holes tend to diffuse laterally, resulting in lateral etching and the formation of pits on the sidewalls. Cold etching temperature is also highly advantageous to reduce the pits on the sidewalls [77]. K. Balasundaram et al. [73] noted that when the etching temperature decreases, the porosity is reduced. R. Akan et al. [35] reported an increase in surface roughness and porosity at the etching temperature of 40 °C.

**Figure 10:**
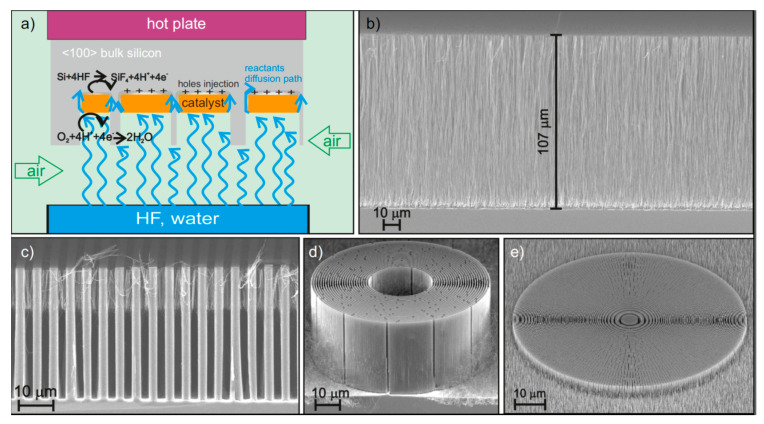
(**a**) Schematic of MacEtch in gas phase. SEM in cross section of structures by gas phase MacEtch at 55 °C, HF was evaporated from a water diluted HF solution and the oxidant is supplied by air: (**b**) Si nanowires; (**c**) linear grating with pitch of 4.8 μm; (**d**) circular grating with pitch 1 μm; (**e**) zone plate with outmost pitch of 200 nm. The figure was adapted with permission from L. Romano, 2020. [37].

Another way to observe the effect of temperature is MacEtch in gas phase [37,78], where the HF is delivered in vapor phase and the oxidant in gas phase (oxygen from air) to the metal patterned Si substrate. It has been recently demonstrated that etching in the vapor phase avoids the issues related to wet-etching such as the nanostructures stiction due to capillary effects during liquid drying. Moreover, the pattern transfer from the metal mask to the silicon template is much more precise and defect-less due to the microporosity reduction and the extremely high concentration of HF, which are not accessible in wet-etching. By increasing the temperature in the range 35–40 °C, the etching rate increases in agreement with previous studies on MacEtch kinetics in liquid [75]. The etching rate has a maximum at 40 °C (see [37]), then it decreases as a function of temperature, indicating that the reaction rate is limited by the desorption of HF. Some examples of nano- and micro-structures of X-ray optics [37] are reported in Figure 10. The etching was realized by evaporating water diluted HF (50 wt.%) at room temperature and exposing the Pt-patterned Si substrate to the HF vapor and air, the gaseous O_2_ present in the air worked as oxidant for the MacEtch reaction. The Pt-patterned Si substrate is held at 55 °C during the etching in order to avoid the moisture’s condense, so the MacEtch reaction temperature is 55 °C and the reaction is considered to happen with a solid–gas interface.

The use of gas-MacEtch turned out to be very useful to improve the stability of free-standing Si nanostructures such as the nanowires and the zone plate in the outmost region. A totally interconnected catalyst design results in free-standing Si nanopillars. As noted by R. Akan et al. [35] for very high aspect ratios and smallest zone sizes, these pillars will become mechanically unstable. Chang et al. [17] and K. Li et al. [36] increased the number of Si interconnects to solve this issue, but this further reduces the active zone plate area and consequently the efficiency. The zone plate made by gas-MacEtch (Figure 10e) does not need Si interconnects since the stability of Si lamellas is not compromised by the liquid drying.

Moreover, nanowires can be used as diffractive optics in speckle based X-ray phase contrast imaging [79]. Nanowires are expected to improve the sensitivity by producing speckles of smaller size in comparison to sandpaper [80] or other membranes with feature size in the micrometer scale.

## 4. Silicon Based Microfabrication of X-ray Optics

Microstructures constitute the X-ray optical elements such as diffractive and refractive X-ray lenses for microfocusing applications at synchrotron beam lines [81] and diffractive gratings for interferometric systems [9,82]. For low energy applications (<10 keV) microfabrication can be realized in silicon but higher X-ray absorbing materials are necessary for hard X-rays. The most common microfabrication approach is based on creating a low X-ray absorbing template and filling it with highly absorbing metal. Historically, the templates are based either on polymer [83,84] or silicon [85].

Silicon microfabrication has been the key technology in manufacturing integrated circuits and microchips in the semiconductor industry. This gives the advantage of a well-assessed technology with a competitive mass production such as deep reactive ion etching [86] and KOH wet etching [87,88]. The combination of unconventional processing and the freedom from microelectronics constrains enrich the spectrum of capabilities and give a new life to the “old silicon material”, with revolutionizing advancements in nanotechnology [37]. We recently reported about micro- and nano-fabrication processing for X-ray gratings, including lithography [89], dry [37,90,91] and wet etching methods [39], Au electroplating [22], Ir atomic layer deposition [92] and metal casting [19,20]. The use of MacEtch as a microfabrication process for X-ray optical devices was first reported in 2014 for Fresnel zone plate structures [17,18]. Some SEM images of Fresnel zone plate structures produced by MacEtch are reported in Figure 3; Figure 10. The X-ray nanofocusing effect of Fresnel zone plate optics fabricated by MacEtch and atomic layer deposition of Pt was recently reported by K. Li et al. [93]. In the following section we report an example of X-ray grating interferometry with gratings fabricated by MacEtch. The main challenge for X-ray grating interferometry is the fabrication of the absorption gratings [85], which are metal periodic microstructures, for high energy X-ray (>30 keV).

Deep X-ray lithography (also called LIGA) [83,84] is used to pattern the polymer template. This technology has the advantage that the polymer pattern can be created on whatever substrate, such as a metallic substrate that is used as a seed layer for the following Au electroplating process in order to create the final Au absorbing grating. The metal layer can be deposited on graphite that has the advantage of being flexible and allows to easily bend the Au grating structure. However, LIGA process is limited to relatively small area (10 × 10 cm^2^) and it is quite expensive since it requires a synchrotron facility. In the case of Si based technology, the Si etched structure can affect the quality of the Au electroplating filling, some distortions [22] or voiding inside the template, resulting in less X-ray diffraction efficiency. Atomic layer deposition of metallic coating has been implemented to create a metallization layer for Au electroplating with conventional damascene approaches [94,95] and Au bottom up superfilling processes [96]. MacEtch offers the possibility to benefit of the original catalyst layer as a seed for the Au electroplating filling.

### Gratings Fabrication for X-ray Phase-Contrast Imaging

X-ray grating interferometry (GI) based imaging is a very promising, fast growing and competitive technique for medical, material science and security applications [1]. Contrast in X-ray imaging with GI can be boosted by exploiting refraction and scattering, in addition to conventional absorption. GI might have a large impact on the radiological approach to medical X-ray imaging because it will intrinsically enable the detection of subtle differences in the electron density of a material (like a lesion delineation) and the measurement of the effective integrated local small-angle scattering power generated by the microscopic structural fluctuations in the specimen (such as micro-calcifications in a breast tissue for instance). Similar enhancements are expected in homeland security or material science application, where structural properties such as orientation, degree of anisotropy, average structure size, and distribution of structural sizes can be inferred via omnidirectional tensor tomography [97].

**Figure 11:**
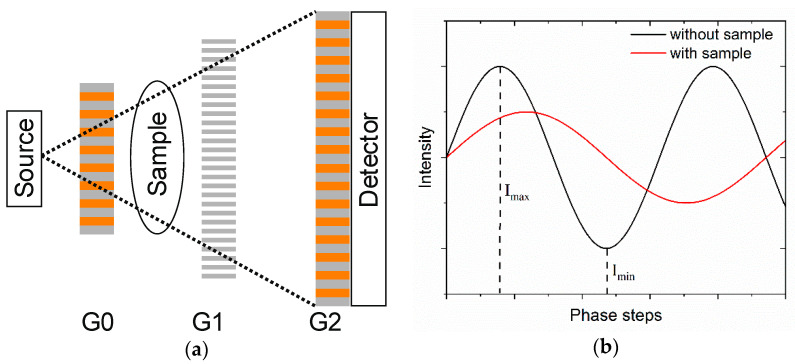
(**a**) Sketch of an X-ray grating interferometer in the Talbot–Lau configuration. (**b**) Scheme of the phase stepping process.

The purpose of an X-ray interferometer is to encode propagation-induced phase changes in the beam wavefront—when passing through a specimen—into an intensity modulation measured by a (usually position sensitive) detector placed downstream. In its simplest configuration, called Talbot Interferometer, an X-ray interferometer consists of two gratings placed in a partially coherent beam. The latter is usually provided by a third/fourth generation synchrotron source or, with significantly less intensity, by a microfocus X-ray tube, see Figure 11. The first grating G_1_ (of period *p*_1_) is usually a phase-grating, i.e., it actually does not absorb the beam but it imposes a significant phase shift resulting in a controlled wavefront modulation at a specific distance downstream, usually where the second, absorbing grating G_2_ (of period *p*_2_) is placed. G_1_ essentially divides the incoming beam into the two first diffraction orders: being the grating pitch (*p*_1_~µm) much larger than the incoming wavelength (~Å), the resulting angle between both diffracted beams is so small that they almost fully overlap, resulting in a linear periodic interference fringe pattern downstream of G_1_, in planes perpendicular to the optical axis. This effect is known as the fractional Talbot effect [98]. A sample of interest is placed either in front or behind G_1_, and it usually absorbs, refracts and scatters the incoming beam. These interactions consequently affect the interference pattern: absorption leads to an average intensity reduction, refraction causes a lateral displacement of the fringes and scattering reduces the fringe amplitude. For a phase grating with a phase shift of π illuminated by a plane wave, the periodicity of the fringe pattern equals *p*_1_/2 [9]. The detector resolution might not be good enough to resolve the interference pattern and therefore a second, absorbing grating G_2_ (with the same periodicity as the fringes) is placed immediately in front of the detector at the position where the fringes form. This grating behaves as a transmission (analyzer) mask and converts local fringe positions into signal intensity variations. This is a crucial aspect of GI, as the analyzer gratings *de-facto* decouples the phase sensitivity of the system from its intrinsic spatial resolution, making GI suitable for operation on large samples and large field of views. In fact, when the source does not provide a sufficiently high spatial coherence, like in the case of a conventional X-ray tube, a third grating G_0_ of period *p*_0_ can be introduced right after the source yielding to the so-called Talbot-Lau (Figure 11a) configuration. G_0_ is an absorbing structure that creates an array of individually coherent, but mutually incoherent sources. If the condition *p*_0_ = *p*_2_ × L_01_/L_12_ is fulfilled [85], where L_01_ is the distance between G_0_ and G_1_ and L_12_ is distance between G_1_ and G_2_, then the images created by each line source are superimposed in the image plane. This enables to carry out efficient phase contrast X-ray imaging on commercially, normally incoherent sources. Retrieval of the absorption, phase, and scattering signals has been done with various methods, with the phase-stepping [9] and the fringe scanning [99,100] being the most common used approaches.

The intensity modulation of the recorded fringe pattern is usually characterized by its visibility (Equation (5)):

(5) $$V=\frac{I_{max}-I_{min}}{I_{max}+I_{min}}$$

where *Imax* and *Imin* are indicated in the phase step curve of Figure 11b. The visibility depends on the degree of spatial coherence of the illumination as well as its spectrum, on the system geometry and on grating’s pitch and depth. The interference fringe visibility is a common figure of merit for the design of X-ray gratings interferometers [101]. This is because the formation of high-modulation fringe pattern is a prerequisite for robust grating interferometry.

**Figure 12:**
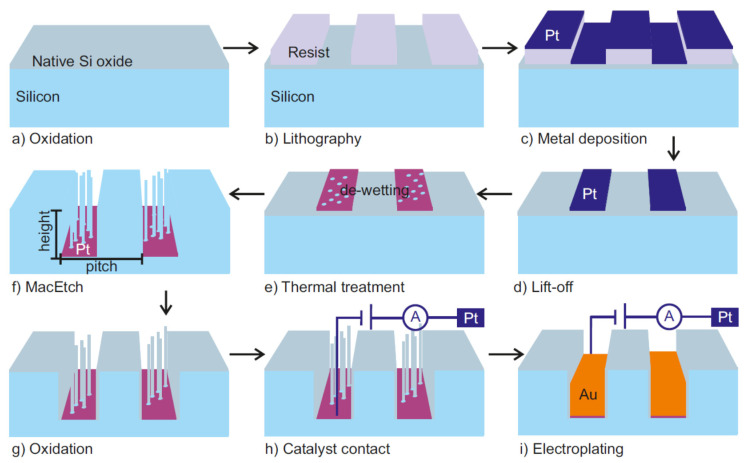
Schematic illustration of the grating fabrication process by MacEtch and subsequent Au electroplating: (**a**) growth of native silicon oxide on Si substrate, (**b**) pattern definition by means of a lithographic process, (**c**) Pt deposition by evaporation, (**d**) lift-off, (**e**) Pt de-wetting by thermal treatment, (**f**) MacEtch in a solution of HF and H_2_O_2_, (**g**) Si side wall oxidation in air, (**h**) electrical contact of the catalyst metal interconnected pattern to the electroplating electrode, (**i**) seeded Au growth by electroplating. The Pt pattern works as a catalyst for MacEtch and as a seed layer for Au electroplating. The figure was adapted with permission from L. Romano et al., 2020. [21].

The main challenge is the fabrication of the absorption gratings [85], which are metal periodic microstructures with high aspect ratio that are usually fabricated starting from templates produced by LIGA [84] or deep Si etching [22,39,85]. The period *p*_2_ (*p*_0_) of the absorbing grating G_2_ (G_0_) is usually in the range of 1–20 µm (5–100 µm), while the height (h) depends on X-ray energy and absorption efficiency of the material [102]. A transmission of the structures of less than 25% is acceptable. It can be calculated [102] that 10 µm thickness is sufficient for photon energies below 20 keV, while for photon energies of 30 keV (60 keV), about 25 µm (160 µm) of gold is required. Very challenging, medically oriented projects, require the design of very sensitive interferometers extending on short geometries, imposing quite extreme aspect-ratios for G_2_ for which periods *p*_2_ as small as 1 µm (or below) and gold height of 30 µm might be needed [103]. Such requirements pushed the research efforts toward MacEtch as a new technique that is able to provide aspect ratio structures with period ranging from tens of nanometers to tens of micrometers. Moreover, MacEtch offers the possibility to benefit of the original catalyst layer as a seed for the Au electroplating filling. Figure 12 summarizes the Si template fabrication by using MacEtch and the subsequent Au electroplating.

**Figure 13:**
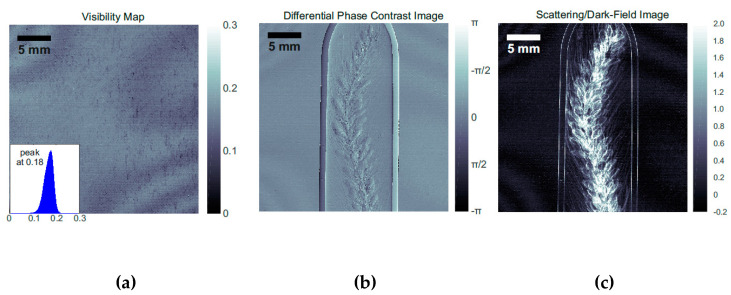
X-ray performance of an Au electroplated grating of 6 µm period used as a G_0_ absorbing grating. X-ray fringe visibility map and visibility histogram as insert (**a**), differential phase contrast (**b**), and scattering (**c**) images of a grain ear. The figure was adapted with permission from L. Romano et al., 2020. [21].

Absorption gratings are usually fabricated by metal electroplating (typically of Au, which is one of the most efficient absorbing materials for X-rays), into high aspect ratio Si templates. The performance of the Au electroplated grating in terms of uniformity and quality of the filling can be well assessed with an X-ray interferometric set up. Figure 13 reports a typical example of grating characterization. The performance of an Au filled grating with a pitch of 6 µm was investigated with an X-ray interferometer [104] (design energy 20 keV) operated at the 3^rd^ Talbot order with π/2 phase shifting G1 grating. The Pt-MacEtch grating (pitch 6 µm, height 39 µm) filled with Au up to 30 µm was used as G0 grating together with a G1 phase grating made of Si by deep reactive ion etching (DRIE) and a G2 absorbing grating fabricated by DRIE and Au electroplating [22]. The average X-ray fringe visibility of 17.5% (Figure 13a) is comparable to the values achieved in absorbing gratings fabricated by conventional DRIE followed by Au electroplating [22]. Figure 13b,c is examples of images obtained with the X-ray grating interferometer, phase contrast (Figure 13b) enhances the detection of low absorbing textures, while dark field (Figure 13c) highlights the presence of microstructures.

## 5. Conclusions and Perspectives

**Figure 14:**
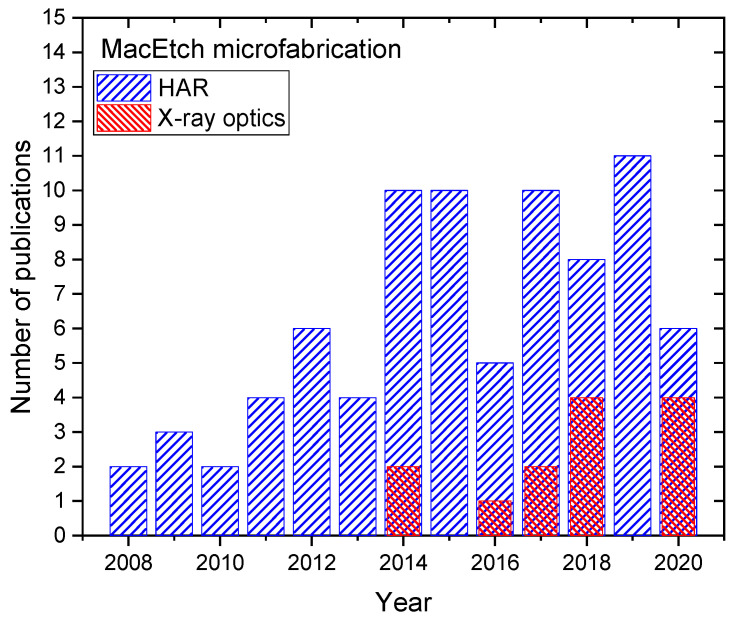
Number of publications as a function of year regarding MacEtch for producing high aspect ratio (HAR) patterned structures [2,8,15,17,18,21,24,25,26,31,32,35,36,37,38,39,40,41,43,47,50,51,54,56,57,58,62,63,71,73,77,78,93,105,106,107,108,109,110,111,112,113,114,115,116,117,118,119,120,121,122,123,124,125,126,127,128,129,130,131,132,133,134,135,136,137,138,139,140,141,142,143,144,145,146,147,148,149,150,151,152,153] and specific for X-ray optics fabrication [17,18,21,35,36,37,38,39,40,41,93,119,126,153].

MacEtch is a very powerful and promising technique that is competing the performances of more conventional etching technology, such as deep reactive ion etching and cryogenic processes [86]. Having a clear idea about the fundamentals and recent advances in this area allows researchers to have a better perspective. During the process of etching, a triggering event that produces unequal etch rates might occur. These events can change the effective forces on the catalyst and produce a resultant torque on the catalyst [51]. Such triggering events can deteriorate the etching uniformity and compromise the quality of the pattern transfer from the original lithography to the Si substrate. We discussed the role of the metal catalyst pattern in order to be able to extent the feature size of the etched structure from nanometers to micrometers and to control the etching uniformity. Metal de-wetting technique turned out to be a reliable method to create a porous catalyst layer that allows to etch very high aspect ratio structures. We discussed the critical role of etching solution by showing the effect of reactants depletion and the presence of alcohol additives. A small amount of alcohol can help to improve the etching uniformity and reduce the formation of the microporosity on the side wall trenches. MacEtch in gas phase showed new possibilities of etching nanostructures with exceptional aspect ratio up to 10,000:1. In the framework of X-ray optics, the fabrication of useful microstructures by MacEtch with successful story of implementations started to record new publications in the last few years, with continuously improving performances. In order to give an idea, we selected all the publications about MacEtch that are relevant to produce high aspect ratio structures in silicon (100) substrates for lithographic patterns such as ordered pillars arrays, electron beam lithographic patterns, gratings, Micro Electro Mechanical Systems (MEMS) microstructures, photonic crystals, etc. Figure 14 reports the number of publications as a function of the publication year to the best of the authors’ knowledge. The total number of publications in the time range of 2008–2020 years is 81, including this special issue of Micromachines, the trend indicates a substantial increment in the last 10 years. The publications addressing the use of MacEtch for specific X-ray optics fabrication is 13 with the first reports in 2014.

X-ray optics with nanostructured features, such as kinoform lenses and Fresnel zone plates, showed to benefit from MacEtch fabrication. We can envisage new future applications of gas-phase MacEtch when high aspect ratio and feature size in the nanoscale are needed, such in X-ray microscopy with energy higher than 20 keV. A full process of gratings microfabrication for X-ray interferometry by using MacEtch and subsequent Au electroplating is demonstrated with performances similar to other fabrication methods. The Pt catalyst layer that sinks down into the Si substrate during MacEtch has been successfully used as seed layer for Au electroplating in order to fabricate a periodic structure with an high absorbing material for hard X-ray. The possibility to push forward the aspect ratio and the relative low fabrication costs on large area of MacEtch with respect to other technologies, such as reactive ion etching and LIGA, are motivating the investigation efforts. We envisage that MacEtch will become a new enabling technology to fulfill the requirements of grating’s height for absorbing hard X-ray radiation and submicron grating’s pitch for boosting the sensitivity in grating based high sensitive X-ray systems, as required for instance for early breast cancer detection.

The main bottleneck in grating based X-ray interferometry is the fabrication of high aspect ratio periodic structures, whose quality and homogeneity over large areas strongly affect the contrast of the generated images. There is the need to produce X-ray diffraction gratings with (i) very high aspect ratio (AR ≥ 50:1) in highly absorbing material such as gold; (ii) large area (mammography, e.g., asks for a field of view above 20 × 20 cm^2^ [103,154]); (iii) good uniformity (no distortions and changes in the duty cycle and depth over the grating area); and (iv) bending capability in order to improve the field of view limitation of cone beam emission from the X-ray source. MacEtch technique has the clear advantage of silicon patterning with high aspect ratio at nanoscale, it is a relatively low cost technology since it is accessible even in labs with limited equipment (no vacuum or clean-room conditions). However, MacEtch is performed in solutions of hydrofluoric acid, we recommend to follow all the necessary safety protocols in order to handle heavily concentrated hydrofluoric acid. The bending capability of the silicon substrate depends on the wafer thickness, extreme bending has been reported for silicon wafers with thickness below 50 µm but the bending of a thick absorbing grating can be challenging and we predict that dedicated processing needs to be developed to avoid cracks and distortions, such as eventually etching off the silicon template. Four inch wafer scale MacEtch gratings have been demonstrated with good uniformity and high control of trench profile and etching direction for aspect ratio up to 30:1 [39]. The process itself has no limits in terms of patterning area such as LIGA, photolithographic processes are available for Si based technology up to 12 inch wafer scale, periodic linear gratings with pitch size in the range of 100 nm can be patterned on wafer scale by interference lithography [89], nanoimprinting processes can be implemented to further increase the patterned area with nanoscale resolution [55]. High quality structures with high aspect ratio require an etching regime that is dominated by the diffusion of the reactive species. Stirring [26] and large volume of solutions [21] are used to homogenize the concentration of reactants during the etching but the effect on etching rate, aspect ratio and defects need to be systematically investigated in order to anticipate the commercialization of MacEtch as a grating fabrication technology. The fabrication throughput as a function of grating quality and performances needs to be assessed to start the technology transfer from the research laboratory level to an industrial R&D.

MacEtch as a technology is still at its infancy, its control and reproducibility over a large area are still not clear and n to be systematically studied. Further scientific efforts need to be made to take full advantage of high aspect ratio capability and exploit its application.
